# A redescripton of *Lyrosoma pallidum* (Eschscholtz) and distributional range extension of *Lyrosoma* Mannerheim (Coleoptera, Agyrtidae)

**DOI:** 10.3897/zookeys.329.4957

**Published:** 2013-09-05

**Authors:** In-Seong Yoo, Derek Sikes, Kee-Jeong Ahn

**Affiliations:** 1 Department of Biology, Chungnam National University, Daejeon 305-764, Republic of Korea; 2 University of Alaska Museum, 907 Yukon Dr., Fairbanks, Alaska, 99775, USA

**Keywords:** *Lyrosoma pallidum*, *L. opacum*, distribution range, coastal, Agyrtidae

## Abstract

A redescription with illustrations of the species *Lyrosoma pallidum* and a key to the Korean species of the family Agyrtidae are provided. New distributional data, including a range extension, of the two *Lyrosoma* Mannerheim species are presented. *Lyrosoma pallidum* (Eschscholtz) is recorded for the first time in Korea.

## Introduction

The genus *Lyrosoma* Mannerheim, containing two species worldwide, is confined to coastal habitats, such as under stones, seaweeds, and carcasses of various coastal animals along the seashore. They have also been reported in nests of maritime birds, but little is known regarding their immature stages and bionomics. They can be recognized by the combination of the following characters: mandibles without subapical teeth; antennomeres 9–10 each with apical grooves including compact distribution of sensilla; elytron with 9 striae; hind wings absent; pro- and mesotarsi dilated in males; aedeagus without parameres (Newton 1997; Schawaller 1998).

We collected a good series of *Lyrosoma pallidum* (Eschscholtz) along the seashores of Korea, Hokkaido (Japan), and Kamchatka (Russia). This species is a new addition to the Korean fauna. Newton (1997) classified *Lyrosoma* under Agyrtinae (one of the three subfamilies, *sensu* Newton 1997) based on abdominal-elytral interacting system and structure of aedeagus, and discussed its phylogenetic relationships with other genera. Later, Schawaller (1998) revised the genus *Lyrosoma*, synonymizing six species, thereby reducing the genus from eight species to only two species, and also reported its distributional range along northern Pacific coasts. However, the description of *Lyrosoma pallidum* was insufficient, lacking important features such as mouthparts and body sculpture and the distributional data were sparse and incompletely mapped. Here, we present a redescription with a habitus photograph and illustrations of *Lyrosoma pallidum*, provide improved distribution data for both species, a range extension for *Lyrosoma pallidum*, and a key to the Korean species of Agyrtidae.

## Material and methods

All *Lyrosoma pallidum* specimens used in this study are deposited in the Chungnam National University Insect Collection (CNUIC), Daejeon, Korea. New data for *Lyrosoma opacum* are from specimens deposited in the University of Alaska Museum Insect Collection (UAM), Fairbanks, Alaska, USA. These data and all literature records reported here for *Lyrosoma opacum* are available online at http://arctos.database.museum/saved/Lyrosoma_opacum. Digital images of habitus were merged using image stacking software (Combine ZP). For measurement, we selected 10 males and 10 females (2♂1♀ from Korea; 5♂6♀ from Japan; 3♂3♀ from Russia) with maximum body size variation. The following abbreviations were used in the text: BL body length (HL+PL+EL); HL length of head from the anterior margin of the clypeus to the posterior margin of head; HW width of head, including the eyes; PL maximum length of pronotum; PW maximum width of pronotum; EL length of elytron from the base to the posterior margin of elytron; EW width of elytra.

## Results

### Lyrosoma pallidum

(Eschscholtz)

http://species-id.net/wiki/Lyrosoma_pallidum

Figs 1 – 10

Pteroloma pallidum Eschscholtz, 1829: 7.
Lyrosoma ovipenne Lewis, 1893 (synonymy by Schawaller 1998)
Lyrosoma suturale Lewis, 1893 (synonymy by Schawaller 1998)
Lyrosoma chujoi Mroczkowski, 1959 (synonymy by Schawaller 1998)
Lyrosoma ituropense Hlisnikovský, 1964 (synonymy by Schawaller 1998)

#### Material.

**KOREA:** Gangwon Prov.: 1♂1♀, Goseong-gun, Hyeonnae-myeon, Daejin-harbor, 38°29'59.1"N, 128°25'46.2"E 9 m, 29 V 2012, IS Yoo, JH Song, seaweeds on seawall; 1♂ (dissected), Sokcho-si, Daepo-dong, Oeongchi-beach, 38°11'01.3"N, 128°36'31.9"E 21 m, 28 V 2012, IS Yoo, JH Song, under seaweeds; **RUSSIA:** 15♂15♀ (1♂1♀ on slide, 8♂7♀ in 80% EtOH), Kamchatka, Petropavlovsk-kamchatsky, Avacha Bay, 53°01'26.4"N, 158°38'31.3"E, 25 VII 2011, KJ Ahn, IS Yoo, under debris on seashore; 2♂2♀ (7♀ in 80% EtOH), Kamchatka, Petropavlovsk-kamchatsky, Rescue Bay, 52°49'31.8"N, 158°35'51.1"E, 27 VII 2011, KJ Ahn, IS Yoo, seaweeds on rocky shore; 1♀ (in 80% EtOH), Kamchatka, Petropavlovsk-kamchatsky, Rescue Bay, 52°49'31.8"N, 158°35'51.1"E, 27 VII 2011, KJ Ahn, IS Yoo, seaweeds on sandy beach; **JAPAN:** 44♂54♀ (1♂1♀ on slide, 24♂30♀ in 80% EtOH), Hokkaido, Oshima, Minami-kayabe, Ôfuna, 42°00'01.9"N, 140°52'55.4"E, 7 VI 2012, KJ Ahn, IS Yoo, decaying seaweeds/starfish on seawall; 1♂, Hokkaido, Akkeshi, Tokotan, 15 VI 1994, K. J. Ahn, on rock crevice in high tide; 3♂18♀, Hokkaido Prov., Nemuro City, Hamamatsu, 26 IX 2000, H.-J. Kim, M.-J. Jeon, under stones.

#### Redescription.

**Figure 1.**
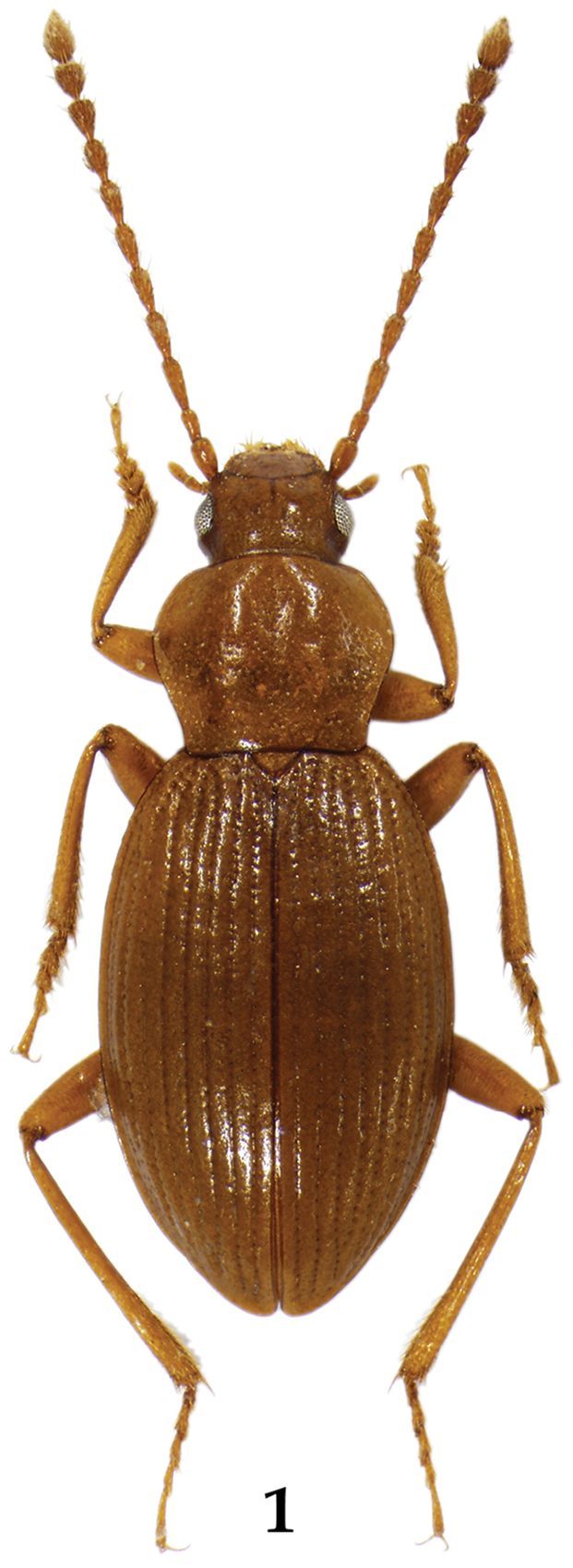
*Lyrosoma pallidum* (male from Goseong, Korea), body length 4.8 mm.

**Figures 2–6.**
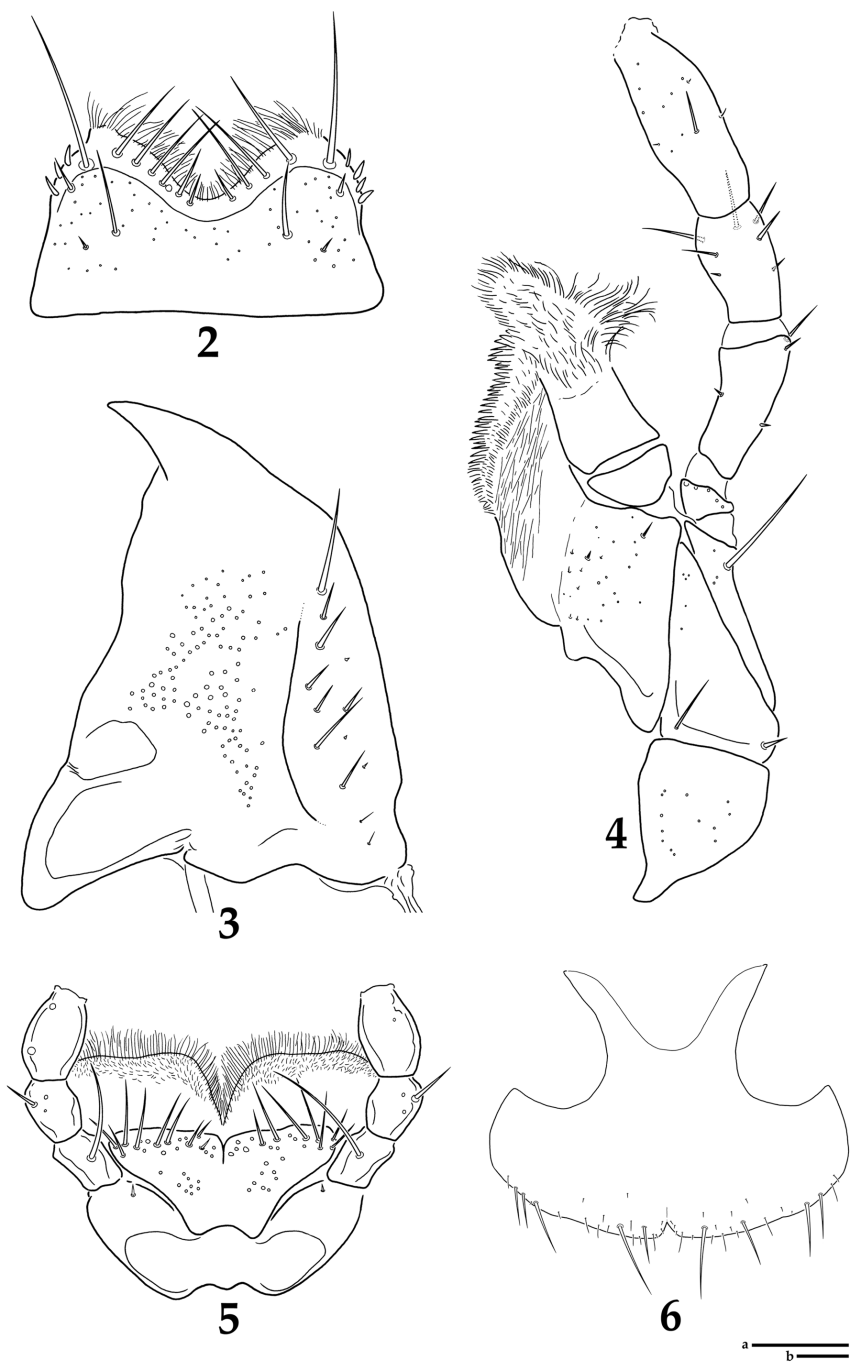
*Lyrosoma pallidum*. **2** labrum, dorsal aspect **3**﻿ right mandible, dorsal aspect **4** left maxilla, ventral aspect **5** labium, ventral aspect **6** female sternite VIII, ventral aspect. Scale bars, 0.1 mm (a: Figs **2–5**; b: Fig. **6**).

**Figures 7–10.**
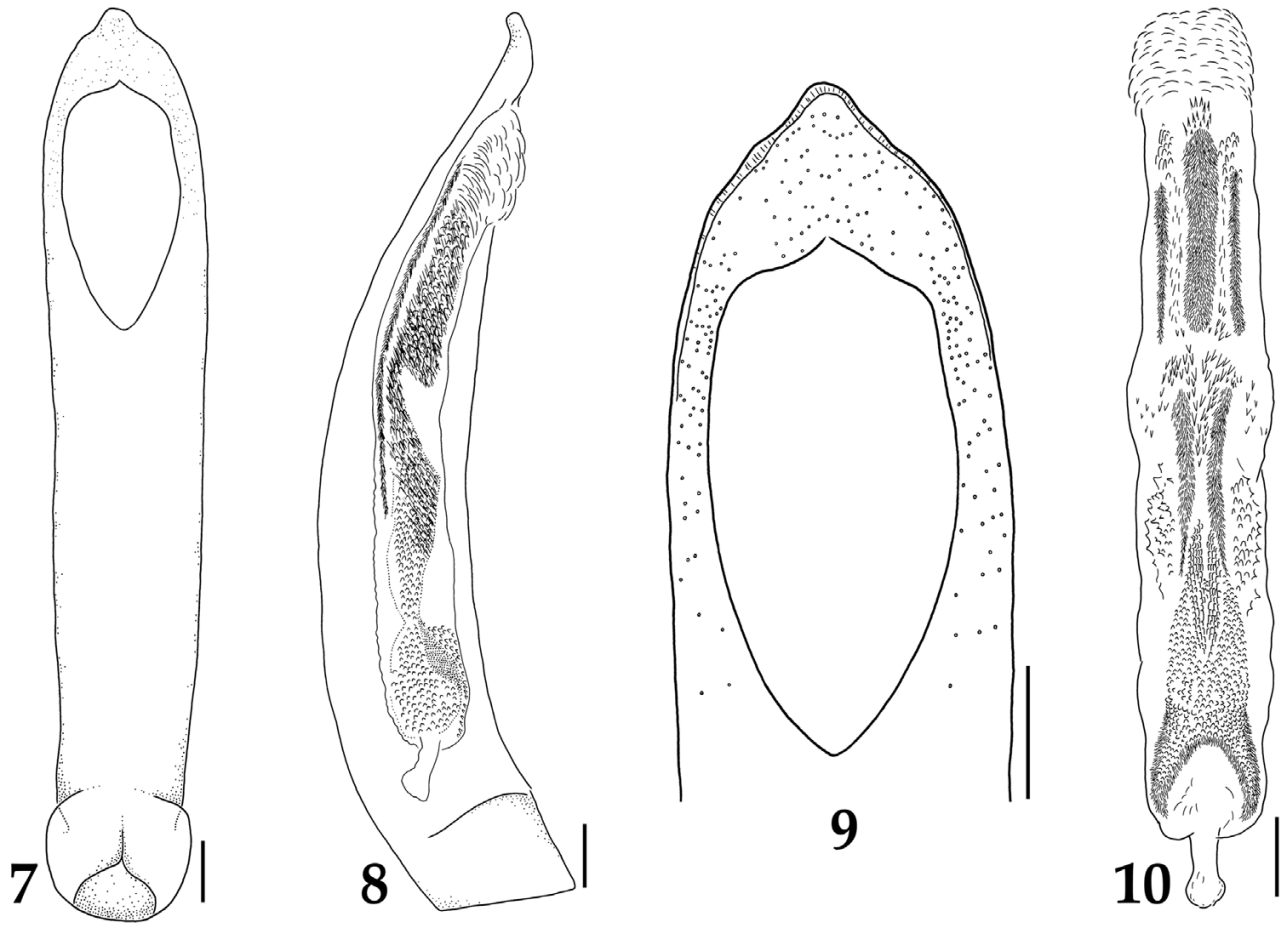
Aedeagus of *Lyrosoma pallidum*. **7** median lobe, ventral aspect **8** median lobe, lateral aspect **9** apex of median lobe, ventral aspect **10** internal sac, ventral aspect. Scale bars, 0.1 mm.

Body (Fig. 1) general carabid-shape, yellowish brown to reddish brown, surface glossy with microsculpture. Male. BL: 4.3–5.1 mm. Head as long as wide or slightly wider than long (HL/HW: 0.94–1.00); eyes medium and prominent, separated by about 5 times of eye width; eye about 0.8 times as long as temple; short setae present between facets, shorter than facet diameter; vertex of head without pale mounds; epistomal suture indistinct; epicranial suture more or less Y-shaped; dorsal surface with rather dense isodiametric microsculpture, ventral surface with transverse microsculpture. Antenna filiform, about 3 times as long as HW, antennomere 2 shortest, 3 and 11 longest, 4–7 similar in shape and length, 9–10 with dense sensilla in apical grooves, 9–11 very weakly clubbed. Labrum (Fig. 2) transverse; anterior margin deeply and broadly emarginate; anterior and each anterolateral margin distinctly membranous and transparent; 4–6 long setae and single very long seta present on membranous part of each side of midline; 3–4 spinose setae present on each anterolateral region of membranous part; anterior margin of membranous part with dense micro setae; sclerotized part with two setae on each side of midline and one seta on each anterior corner; surface of sclerotized part with many pores scattered sparsely. Mandibles (Fig. 3) symmetrical, inner regions without tooth; broad at base and pointed at apex; dorsal and ventral surface with many pores on medial area. Maxilla (Fig. 4) with 4 palpomeres; palpomeres 2–4 with distinct setae. Labium (Fig. 5) with 3 palpomeres; palpomeres 1–2 with long setae, last palpomere asetose; ligula caudal fin-like shape and bilobed, each lobe with 7–10 long setae on subapical region; ligula with many pores on medial and subapical regions of each lobe; paraglossa broadly developed, anterior margin with many setae densely distributed. Mentum trapezoidal narrowing apically; anterior margin straight; single long seta present on each anterolateral region; many pores scattered sparsely and microsculpture transverse. Submentum with many pores, short setae present sparsely. Pronotum broad cordiform, wider than long (PL/PW: 0.81–0.84); pronotum widest near anterior third; anterior corners round, posterior corners angled; pronotum without mid-basal fovea; disc with shallow and broad medial-longitudinal depression. Elytra oval and convex, gradually increased to posteriorly in convexity; longer than wide (EL/EW: 1.41–1.59); each elytron with 9 striae; intervals as wide as width of first antennomere, weakly convex and impunctate, dense isodiametric microsculpture present; basal lateral margin weakly serrate; epipleura without distinct punctures. Hind wings absent. Mesoventrite glabrous and sculptured strongly; mesoventral process sharp, contiguous to metaventral process. Metaventrite covering many setae sparsely, metaventral process sharp. Pro- and mesotarsi dilated, tarsomere 1−4 with numerous setae ventrally. Metatarsi simple and long; last tarsomere about 1.8 times as long as tarsomere 4. Tarsal claws simple, basal part with very feeble tooth; two empodial setae present, as long as tarsal claws. Abdominal sternites covering many setae, surface with dense isodiametric microsculpture. Aedeagus (Figs 7–10) without paramere; median lobe long and slender, middle part of apex slightly protruded (Fig. 9); structure of internal sac as in Figs 8 and 10. Female. BL: 4.6–5.2 mm. Basically not differ to male. Pro- and mesotarsi not dilated. Sternite VIII (Fig. 6) with posterior margin emarginate narrowly, a few setae present on marginal region; spiculum ventrale broad, deeply emarginate anteriorly.

#### Distribution.

**Figure 11.**
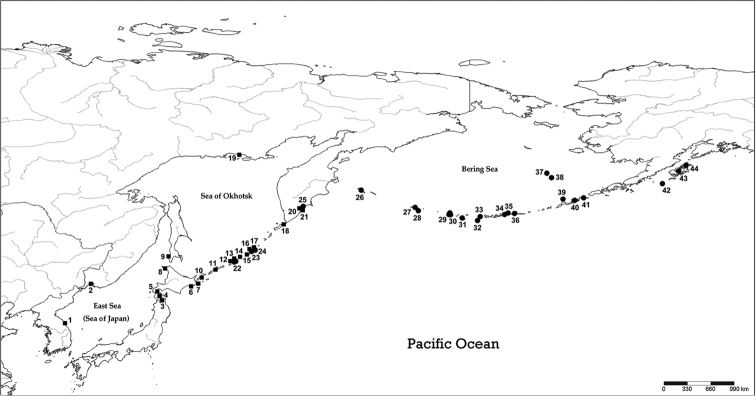
Distribution map of *Lyrosoma pallidum* (solid square, 1–21) and *Lyrosoma opacum* (solid circle, 22–44). **1 Korea: Gangwon Prov.: Goseong, Sokcho 2** Russia: Chasan: Barabasch **3** Japan: Honshu: Aomori **4** Japan: Hokkaido: Hakodate **5 Japan: Hokkaido: Oshima 6 Japan: Hokkaido: Akkeshi, Nemuro 7** Japan: Hokkaido: Cape Nosappu **8** Japan: Hokkaido: Rishiri-tô Island **9** Russia: Sakhalin: Pravda **10** Russia: Kurile Island Kunashir **11** Russia: Kurile Island Iturup **12** Russia: Kurile Island Urup **13** Russia: Kurile Island Cirpoi **14** Russia: Kurile Island Brouton **15** Russia: Kurile Island Simushir **16** Russia: Kurile Island Ketoi **17** Russia: Kurile Island Yanchika **18** Russia: Kurile Island Shumshu **19** Russia: Magadan **20** Russia: Kamchatka: Petropavlovsk **21 Russia: Petropavlovsk-Kamchatsky 22** Russia: Tairu nr Urup / Kurile Island Cirpoi **23** Russia: Kurile Island Ketoi **24** Russia: Kurile Island Yanchika **25** Russia: Kamchatka coast **26** Russia: Bering Island **27** United States: Attu Island **28** United States: Agattu Island **29 United States: Kiska Island 30** United States: Little Kiska **31** United States: Amchitka Island **32 United States: Amatignak Island 33** United States: Gareloi Island **34 United States: Ulak Island 35** United States: Kasatochi Island **36** United States: Atka Island **37** United States: St. Paul Island **38** United States: St. George Island **39** United States: Bogoslof Island **40** United States: Unalaska Island **41 United States: Rootok Island 42 United States: Chirikof Island 43** United States: Kodiak Island **44** United States: Afognak Island. **Bold** records: present study; others: previous distributional sites based on Hamilton 1894; Van Dyke 1921; Hatch 1938; Mroczkowski 1959; Shibata 1969; Nishikawa 1997; Schawaller 1998; Sikes and Slowik 2010.

Korea (new record), Russia (Kamchatka, Kuriles, Magadan, Sakhalin) and Japan (Hokkaido, Honshu), Figure 11.

### Lyrosoma opacum

Mannerheim

http://species-id.net/wiki/Lyrosoma_opacum

Lyrosoma snowi Lewis, 1893 (synonymy by Schawaller 1998)
Lyrosoma tripartitum Lewis, 1893 (synonymy by Schawaller 1998)

#### New records.

See also http://arctos.database.museum/saved/Lyrosoma-opacum-new. **USA: Alaska**: 1 ex. Amatignak Isl., Ulva Cove, 3.9624m el. 51.26002°, -179.07718° ±5m ryegrass pebble beach, pitfall, 6JUN-31JUL 2009 D.S. Sikes; 16 ex. Bogoslof Isl., 1-44m el. 53.9308°, -168.03633° ±720m 16-17JUL 2008 D.M. Collet; 2 ex. Chirikof Isl, 55.82461°, -155.73598° ±3km back beach, hand, 30MAY-3JUN 2012 J.J. Withrow; 1 ex. Kasatochi E coast, upper shore, 52.17165°, -175.49229° ±2m under algae, 18JUN 2010 J. Slowik 1430-1515; 1 ex. Kasatochi, 1.524m el. 52.15905°, -175.4993° ±5m under kelp wrack, 13JUN 2009 J. Williams; 5 ex. Kasatochi, 12m el. 52.17177°, -175.52917° ±11m under rocks, scree, & grass, hand collected, 11JUN 2008 D.S. Sikes; 16 ex. Kasatochi, 3m el. 52.17912°, -175.49708° ±5m above beach, 12JUN 2009 J. Williams; 20 ex. Kasatochi, 3m el. 52.1796°, -175.49722° ±5m bird carcass, 12AUG 2009 D.S. Sikes; 8 ex. Kasatochi, 4.572m el. 52.17912°, -175.49708° ±5m above beach, under crested auklet carcass, 12JUN 2009 J. Williams; 2 ex. Kasatochi, E side, 18m el. 52.17416°, -175.49448° ±25m Peregrine falcon nest, carcasses of prey, 11AUG 2010 J. Williams; 2 ex. Kasatochi, E side, 4m el. 52.17371°, -175.49289° ±5m bird carcass, cliff base, 11AUG 2010 D.S. Sikes; 1 ex. Kasatochi, NE coast, 4m el. 52.18058°, -175.49878° ±8m fish carcass, back beach, 11AUG 2010 D.S. Sikes; 1 ex. Kiska Harbor, 3m el. 51.97823°, 177.53963° ±7m beach cave, 3m deep, 6AUG 2010 D.S. Sikes; 2 ex. Kiska Is., Sirius Pt., 15m el. 52.13293°, 177.59668° ±15m sand, *Honkenya peploides*, 5 pitfalls, 2-15JUL 2008 A. Bond; 5 ex. Rootok, 1m el. 54.05005°, -165.5112° ±5m in cracks back beach rocks, 12JUL 2009 D.S. Sikes; 1 ex. Ulak Isl., 1-160m el. 52.04187°, -175.90281° ±800m *Elymus* stand, 20JUL 2008 D.M. Collet.

#### Distribution.

Russia (Kurile Islands from Urup northwards, Kamchatka, Commander Islands), United States (Aleutian, Pribilofs, Kodiak, Afognak, and Chirikof islands), Figure 11.

## Discussion

The coastal genus *Lyrosoma* includes only two valid species. *Lyrosoma pallidum* can be distinguished from *Lyrosoma opacum* by its smaller body size, absence of epistomal suture and an elytral disc without distinct reticulation (Schawaller 1998). While examining five specimens on slides, we discovered variation in the number of setae on the labrum and ligula of *Lyrosoma pallidum*. These features we consider to be intraspecific variation.

Schawaller (1998) reported that the genus *Lyrosoma* has been recorded on the northwestern Pacific coasts, from the western Aleutian Islands to the southernmost parts of northern Honshu, Japan. Our collection revealed that the distributional range of this genus extends to South Korea, representing a new southernmost boundary (Fig. 11). Additionally, although Schawaller (1998) reported records for *Lyrosoma opacum* from Kodiak and Afognak islands, Alaska, based on citations of Lafer (1989) and specimens in the Natural History Museum (London), he excluded these records from his map and his summary of the distribution of this species. Along this species’ eastern range Schawaller described its distribution as restricted to the western Aleutians and just one of the two Pribilof islands (St. Paul). We have corrected this exclusion and based on our collections (n=6) and literature records not included in Schawaller (1998) (n=17) increased the number of islands on which *Lyrosoma opacum* is known from the seven reported in Schawaller (1998) to twenty-three (Fig. 11). The two *Lyrosoma* species are apparently sympatric on Kamchatka and on the Kurile Islands Ketoi, Cirpoi, and Yanchika.

### Key to the known species of Korean Agyrtidae

The key is modified from Newton (1997), Cho et al. (2001), and Lafer (2002).

**Table:** 

1	Antennomeres 2–5 each with apical grooves including compact distribution of sensilla (see Newton 1997: fig. 3); mandibles without subapical teeth [Agyrtinae]; seashore habitats	*Lyrosoma pallidum* (Eschscholtz)
–	Antennomeres without apical grooves, sensilla sparsely present at the tip (see Newton 1997: fig. 41); mandibles with large subapical teeth [Pterolomatinae]; terrestrial habitats	2
2	Vertex of head without mounds analogous to ocelli; tibiae not carinate; aedeagus without parameres	*Apteroloma kozlovi* Semenov & Znojko
–	Vertex of head with one pair of mounds analogous to ocelli; tibiae carinate dorsally; aedeagus with parameres	3
3	Pronotum cordate, widest at anterior third, dense punctures present mostly along marginal region; aedeagus as in fig. 2F (Lafer 2002)	*Pteroloma forsstromii* (Gyllenhal)
–	Pronotum transverse, widest at the middle, dense punctures present excluding small part of central region; aedeagus as in figs 2A–B (Cho et al. 2001)	*Pteroloma koebelei* Van Dyke

## Supplementary Material

XML Treatment for Lyrosoma pallidum

XML Treatment for Lyrosoma opacum

## References

[B1] Cho YB Park SJ Ahn KJ (2001) A taxonomic review of Agyrtidae (Insecta, Coleoptera) in Korea. The Korean Journal of Systematic Zoology 17(2): 217-222

[B2] Hamilton J (1894) Catalogue of the Coleoptera of Alaska, with the synonymy and distribution. Transactions of the American Entomological Society 21: 1-38

[B3] Hatch MH (1938) Report on the Coleoptera collected by Dr. Victor B. Scheffer on the Aleutian Islands in 1937. The Pan Pacific Entomologist 14(4): 145-149

[B4] Lafer GSh (1989) Family Silphidae (in Russian) Opred. Nasek.Dal’nego Vostoka SSSR 3: 329–344

[B5] Lafer GSh (2002) A review of species of the genus *Pteroloma* Gyllenhal (Coleoptera: Pterolominae) from the Russian Far East with the description of a new species from the south of Primorsky Kray. Baltic Journal of Coleopterology 2(1): 49-61

[B6] Mroczkowski M (1959) *Lyrosoma chujoi* sp. n. from Japan (Col., Silphidae). The Entomological Review of Japan 10(2): 49-50

[B7] Newton AF Jr. (1997) Review of Agyrtidae (Coleoptera), with a new genus and species from New Zealand. Annales Zoologici, Warszawa, 47(1/2): 111–156

[B8] Nishikawa M (1997) *Lyrosoma ituropense* Hlisnikovský (Coleoptera, Agyrtidae) from Hokkaido, North Japan. Elytra 25(1): 121-122

[B9] Schawaller W (1998) Revision of the genus *Lyrosoma* Mannerheim, 1853 (Coleoptera, Agyrtidae). Entomologische Blätter 94: 127-133

[B10] Shibata T (1969) Some reports on the burying beetles from Japan, I (Col., Silphidae). The Entomological Review of Japan 21(2): 47-54

[B11] Sikes DS Slowik J (2010) Terrestrial arthropods of pre- and posteruption Kasatochi Island, Alaska, 2008-2009: A shift from a plant-based to a necromass-based food web. Arctic, Antarctic and Alpine Research 42: 297-305.10.1657/1938-4246-42.3.297

[B12] Van Dyke EC (1921) Coleoptera from the Pribilof Islands, Alaska. Proceedings of the California Academy of Sciences. 4th Ser. XI (14): 156–166

